# Evaluation of an Anesthesia Dashboard Functional Model Based on a Manufacturer-Independent Communication Standard: Comparative Feasibility Study

**DOI:** 10.2196/12553

**Published:** 2019-05-01

**Authors:** Marian Ohligs, Carina Pereira, Verena Voigt, Marcus Koeny, Armin Janß, Rolf Rossaint, Michael Czaplik

**Affiliations:** 1 Department of Anesthesiology, Faculty of Medicine Rheinisch-Westfälische Technische Hochschule Aachen University Aachen Germany; 2 Chair of Medical Engineering, Faculty of Mechanical Engineering Rheinisch-Westfälische Technische Hochschule Aachen University Aachen Germany

**Keywords:** operating room, anesthesia, interconnection, networking, human-computer interaction, process optimization, intelligent alarms, decision-support systems, 11073 SDC, service-oriented device connectivity

## Abstract

**Background:**

Current anesthesia workspaces consist of several technical devices, such as patient monitors, anesthesia machines, among others. Commonly, they are produced by different manufacturers; thus, they differ in terms of their modus operandi, user interface, and representation of alarms. Merging the information from these devices using a single joint protocol and displaying it in a single graphical user interface could lead to a general improvement in perioperative management. For this purpose, the recently approved and published Institute of Electrical and Electronics Engineers 11073 service-oriented device connectivity standard was implemented.

**Objective:**

This paper aims to develop and then evaluate an anesthesia workstation (ANWS) functional model in terms of usability, fulfillment of clinical requirements, and expected improvements in patient safety.

**Methods:**

To compare the self-developed ANWS with the conventional system, a pilot observational study was conducted at the University Hospital Aachen, Germany. A total of 5 anesthesiologists were asked to perform different tasks using the ANWS and then the conventional setup. For evaluation purposes, response times were measured and an interaction-centered usability test with an eye-tracking system was carried out. Finally, the subjects were asked to fill in a questionnaire in order to measure user satisfaction.

**Results:**

Response times were significantly higher when using the ANWS, but decreased considerably after one repetition. Furthermore, usability was rated as excellent (≥95) according to the System Usability Scale score, and the majority of clinical requirements were met.

**Conclusions:**

In general, the results were highly encouraging, considering that the ANWS was only a functional model, as well as the lack of training of the participants. However, further studies are necessary to improve the universal user interface and the interplay of the various networked devices.

## Introduction

In recent decades, the development of new innovative medical devices has led to significant improvements in patient safety, quality of supply, and economic advantages. Conventional anesthesia workspaces, for instance, consist of many different technical devices, including (1) a patient monitor, which records and displays the patient's vital parameters, such as electrocardiogram, body temperature, blood pressure, oxygen saturation, pulse rate, and respiratory rate; (2) an anesthesia machine, which is used to support the administration of anesthesia and for mechanical ventilation; and (3) syringe pumps, which administer intravenous anesthesia. Nowadays, all of these devices are the basis for standard patient care in anesthesiology [1].

However, medical devices assembled for clinical applications are usually produced by different manufacturers. They vary in terms of their modus operandi, user interface, and representation of alarms [2,3]. In intensive care, which represents an example of a data-rich environment, studies have demonstrated that 80% of user errors are a result of cognitive overload [4]. Medical device interoperability allows the joining of all data sources and, thus, leads to a unified presentation and control. To date, there is a lack of a single, shared communication protocol as well as a common interface between devices from different manufacturers [5]. This is especially true among intelligent decision-support systems [6], monitoring systems, and supervision systems [7], which are becoming more and more relevant in modern clinical practice. The fusion of all of this important information (eg, patient data and system settings) in a single graphical user interface (GUI) would simplify the work of anesthesiologists and improve patient safety [8], for example, to better determine the depth of anesthesia and to better optimize perioperative management, including logistics [9,10]. In addition, current patient data management systems only collect information from a number of devices if a proprietary connector is available.

In order to solve the issues discussed, an interdisciplinary consortium composed of engineers, computer scientists, and physicians from approximately 50 German organizations (ie, research institutes, hospitals and clinics, and medical companies) initiated the Secure Dynamic Networking in the Operating Room and Clinic (OR.NET) research project, which was funded by the German Federal Ministry of Education and Research (grant number 16KT1238). Two of the main goals were to develop a single and sophisticated protocol—the service-oriented device connectivity (SDC) family of standards, formerly the Open Surgical Communication Protocol—for medical device communication, as well as a new anesthesia workstation (ANWS), focusing at the same time on human-computer interaction (HCI) and safety concepts [11]. The SDC standards were developed as a general peer-to-peer interconnection protocol for an accurate exchange of medical information (eg, vital parameters and alarms) within operating room networks. They were based on the Device Profile for Web Services standard [12,13], which ensures that each device can communicate in a service-oriented architecture with the help of standard Web services [14,15]. Standard Ethernet is used for system communication since it is cost-effective and supported by the vast majority of medical devices, allowing manufacturer-independent interconnections [11]. After several years of research, the developed OR.NET architecture was included in the Institute of Electrical and Electronics Engineers (IEEE) 11073-SDC family of standards (Health informatics—point-of-care medical device communication), which address interoperability of medical devices and clinical IT infrastructure [16].

In addition to technical interoperability issues, such as definition of data and communication protocols, safety and risk management is also part of the 11073-SDC communication protocol extensions 10207, 20702, and 20701. They were approved and published by the IEEE in January 2019. The so-called *safety classifications* regularize the responsibility among interconnected medical devices and software services.

**Figure 1:**
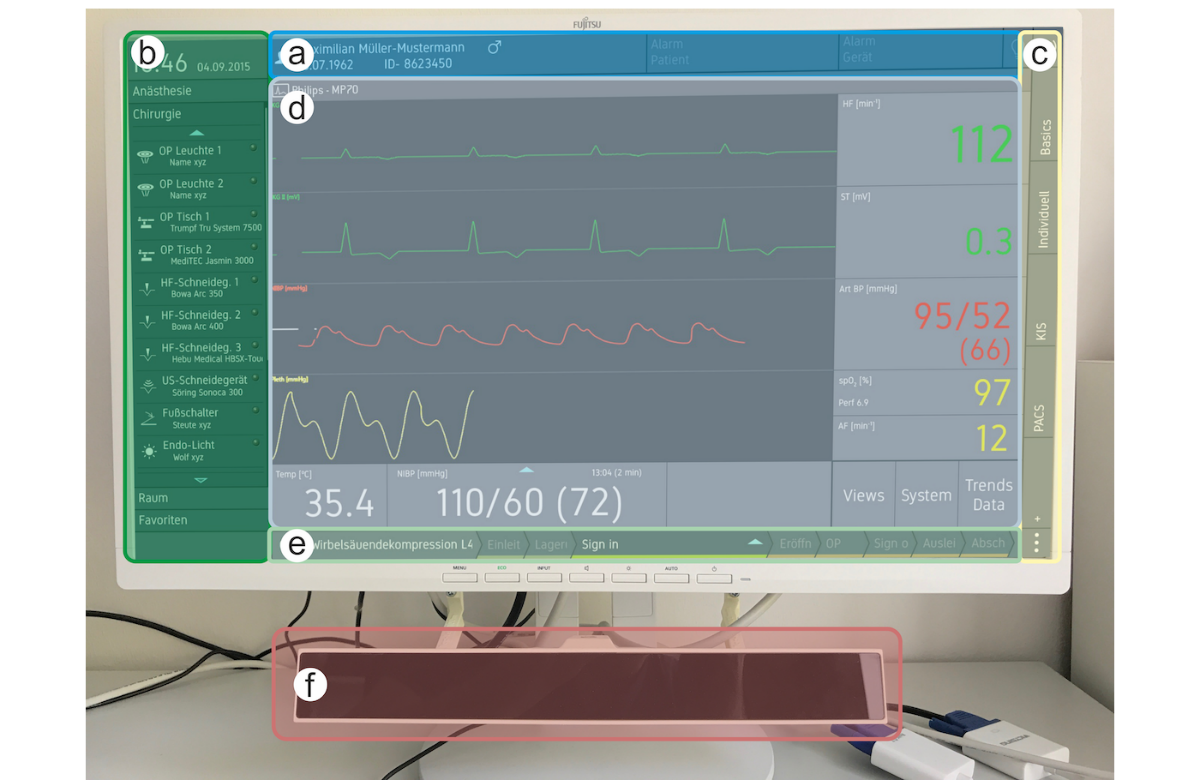
The anesthesia workstation (ANWS) functional model. The interface screen consists of six main elements: (a) patient context and alarms; (b) device panel: overview of all devices connected to the ANWS; (c) view mode selector; (d) area reserved for the selected view; (e) workflow process; and (f) eye-tracking system.

The ANWS functional model was developed to demonstrate and examine the capabilities of medical device interconnections based on the SDC standards; the model is represented in Figure 1. Nonetheless, a common platform presents several advantages in terms of patient safety and teamwork in the operating theater. First, a GUI, which gathers the core information from different sources, offers the physician a better overview of the patient’s overall state as well as of the whole clinical setup and setting. Secondly, interdisciplinary standard operating procedures (SOPs) and checklists (eg, the Surgical Safety Checklist [SSC]) integrated into the ANWS contribute to a better workflow between different medical departments, such as anesthesia and surgery [7,17]. Thirdly, a common platform might minimize the failure rate for documentation, while reducing the physician’s workload as well as the number of nondigital documents [5].

The objective of this paper is to evaluate the ANWS functional model developed during the OR.NET research project in terms of usability, fulfillment of clinical requirements, and expected improvements in patient safety.

## Methods

### Study Design

#### Overview

In order to evaluate the usability of the OR.NET ANWS, and consequently the HCI, a study involving 5 professional volunteers was conducted at the University Hospital Aachen, Germany. Potential benefits of the functional model over a conventional system were examined. Therefore, every participant started with the OR.NET setting, followed by the conventional setup a few weeks later. During a total hip arthroplasty (ie, total hip replacement) surgery simulation, the usability of both systems was evaluated by measuring the usability criteria (ie, effectiveness, efficiency, learnability, and user satisfaction); criteria were measured by (1) using the response time and (2) applying the think-aloud method. Thinking aloud is often used in usability testing by asking the participants to say whatever comes into their mind while performing certain tasks. This method enables insights into the participant’s cognitive processes, including perception, doing, and feeling [18,19]. For the evaluation of the ANWS, further factors based on eye tracking were examined, such as (1) detection of “vampire effects” (ie, when eye catchers draw away and consume the user’s attention) [20]; (2) hidden affordance (ie, when the functionality and intended use of a particular control are not intuitive); and (3) areas of interest [18,19]. At the end of the study, the participants were asked to rate some important features of the ANWS, for example, automatic documentation, compilation of all alarms, and decision-support system.

#### Anesthesia Workstation

##### Overview

The ANWS was installed on a desktop computer with the following properties: Quad-Core i5 CPU, 8 GB RAM, 250 GB solid-state drive, and a Radeon R7 200 dedicated graphics card with 1 GB graphics double-data rate type 5 memory. It was started as a stand-alone executable based on the .NET Framework 4.6 and OpenStackClient C# library (OSCLib C#), version 0.97_09 (SurgiTAIX AG).

##### Simulator

An OR.NET-SDC device simulator (Ilara GmbH) was used during the study to simulate measurements and output data for the following medical devices: syringe pump, patient monitor, and anesthesia machine. Thus, predetermined clinical scenarios could be reproduced. The simulator transmitted scheduled numerical data (eg, heart rate, blood pressure, and oxygen saturation) as well as physiological waveforms (eg, electrocardiography and respiratory curves) inside the network. In addition, it was able to respond to external commands; these were used to set parameters in the simulated devices (eg, infusion rates of syringe pumps). It was started as a stand-alone executable based on Microsoft Office 2016, .NET Framework 4.5, and OSCLib C#, version 0.96_00. The ANWS and the simulator were connected using the SDC through an internal network on the test computer.

##### Eye Tracking

To monitor the users’ behavior (ie, the users’ visual attention on user interface elements), we used an eye-tracking camera system—Gazepoint GP3 HD with Gazepoint Analysis and Gazepoint Control recording software, version 3.5 (Gazept). This was installed below the external monitor, as displayed in Figure 1. Eye-tracking videos and eye-tracking measurements were recorded with approval from the participants. In addition, the desktop window was also acquired using the Gazepoint Analysis software.

##### Audio Acquisition

The subjects were asked to report or express every single thought while solving the tasks (ie, think-aloud method). During the study, an audio record was created for future analysis. Here, the microphone from the LIVE! Cam Chat HD webcam (Creative) was used.

##### Study Tasks

In the first phase, the participants had to carry out 42 tasks. As described in Table 1, a total of 33 tasks were identical for both study phases. To test additional features of the ANWS, nine further tasks were carried out. In general, they consisted of looking up a value displayed on the ANWS as well as setting new parameters, such as flow rate of syringe pumps and positive end-expiratory pressure (PEEP) of the anesthesia machine. Table 1 describes the tasks performed by the subjects during this usability study.

##### Questionnaire

**Figure 2:**
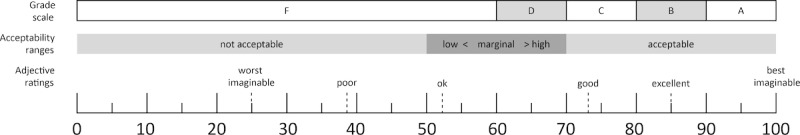
System Usability Scale (SUS) scores. Graphical overview of the adjective ratings, acceptability scores, and school grading scales, in relation to the average SUS score. Figure adapted from Bangor et al [22].

At the end of the study, each participant filled in a survey that included questions about sociodemographic aspects, technical expertise, motivation, study conditions, and features tested. For each tested feature of the ANWS (see Table 2), the System Usability Scale (SUS) developed by John Brooke [21] was used to assess the user’s opinion. To calculate the SUS, 10 specified questions were rated using a 5-point Likert scale ranging from 1 (*strongly disagree*) to 5 (*strongly agree*). Whereas the rates of the positively formulated questions were subtracted by 1, resulting in a score from 0 to 4, the rates of negatively formulated questions were subtracted from 5, also resulting in a score from 0 to 4. Finally, all 10 scores from the questions—five positively formulated and five negatively formulated—were summed and multiplied by 2.5. The results were graded from A to F and compared to acceptability ranges and adjective ratings, as illustrated in Figure 2 [21,22].

**Table 1:** Tasks carried out by the subjects in both study phases.

Task	Study phase	Number of repetitions
Select type of surgery and load respective workflow	ANWS^a^	1
Customize workstation window	ANWS	1
Update workflow process	ANWS	4
Read blood pressure	ANWS, C^b^ (PM^c^)	4
Read temperature	ANWS, C (PM)	3
Read oxygen saturation	ANWS, C (PM)	7
Read heart rate	ANWS, C (PM)	2
Read airway pressure	ANWS, C (AM^d^)	4
Read fraction of inspired oxygen	ANWS, C (AM)	1
Read respiratory minute volume	ANWS, C (AM)	1
Read respiratory compliance	ANWS, C (AM)	1
Set positive end-expiratory pressure (ie, respirator parameter)	ANWS, C (AM)	2
Set infusion flow rate	ANWS, C (SP^e^)	4
Fetch and complete Surgical Safety Checklist	ANWS, C	3
Consider a pulmonary embolism	ANWS, C	1
Switch workspace view	ANWS	2
Check for intelligent alarms	ANWS	1
Total tasks	ANWS, C	42 (ANWS); 33 (C)

^a^ANWS: anesthesia workstation.

^b^C: conventional setting.

^c^PM: patient monitor.

^d^AM: anesthesia machine.

^e^SP: syringe pump.

**Table 2:** Overview of all features integrated into the anesthesia workstation and respective descriptions.

Feature^a^	Tested	Description
Automatic documentation	No	Possibility of saving all information provided by a service-oriented device connectivity-compatible device inside the OR.NET^b^ network, including meta information, through the ANWS^c^.
Compilation of all alarms	No	Alarms provided by any anesthesia-related device (eg, syringe pump, anesthesia machine, and patient monitor) are collected and displayed in the ANWS.
Control devices from other departments	No	Bidirectional control of devices (eg, anesthesiologists can control surgery devices and surgeons can control anesthesia equipment).
Display content from other departments	No	Capability to integrate several or even single measures or parameters in any connected device (eg, to display the current blood pressure, measured by the patient monitor, in the surgical microscope).
Cross-device interaction	Yes	Enables (eg, control of) diverse surgical devices using a single universal footswitch, button, or joystick.
Decision-support system	Yes	Context-adaptive hints and suggestions are displayed based on the currently ongoing surgical intervention, the actual workflow step, eventual patient-related problems, etc.
Segregated alarms	No	All alarms are classified as medical or device-associated (ie, technical) alarms.
Surgical Safety Checklist	Yes	Display of the Surgical Safety Checklist as an integrative part of the surgical workflow.
Unified surface	Yes	Fusion of information from several devices in a single graphical user interface.
Workflow management	Yes	Generation from a workflow based on previous data (ie, database). After each step, the process is updated automatically or manually, enabling significantly improved (ie, predictive) planning.

^a^All features were subjectively rated; five of those were additionally included in a user test scenario.

^b^OR.NET: Secure Dynamic Networking in the Operating Room and Clinic.

^c^ANWS: anesthesia workstation.

Finally, participants were asked to prioritize the tested features according to the relevance in their daily work. Furthermore, they were requested to compare the OR.NET ANWS with common or conventional systems in terms of personal preference and impact on patient safety.

#### Conventional System

In the conventional phase, the subjects were asked to carry out 33 tasks (see Table 1). These consisted of looking up a value displayed on one of the devices (eg, blood pressure, temperature, and heart rate) or of setting one of these parameters (eg, flow rate of syringe pumps or PEEP). Note that these tasks were the same for the OR.NET and the conventional systems.

**Figure 3:**
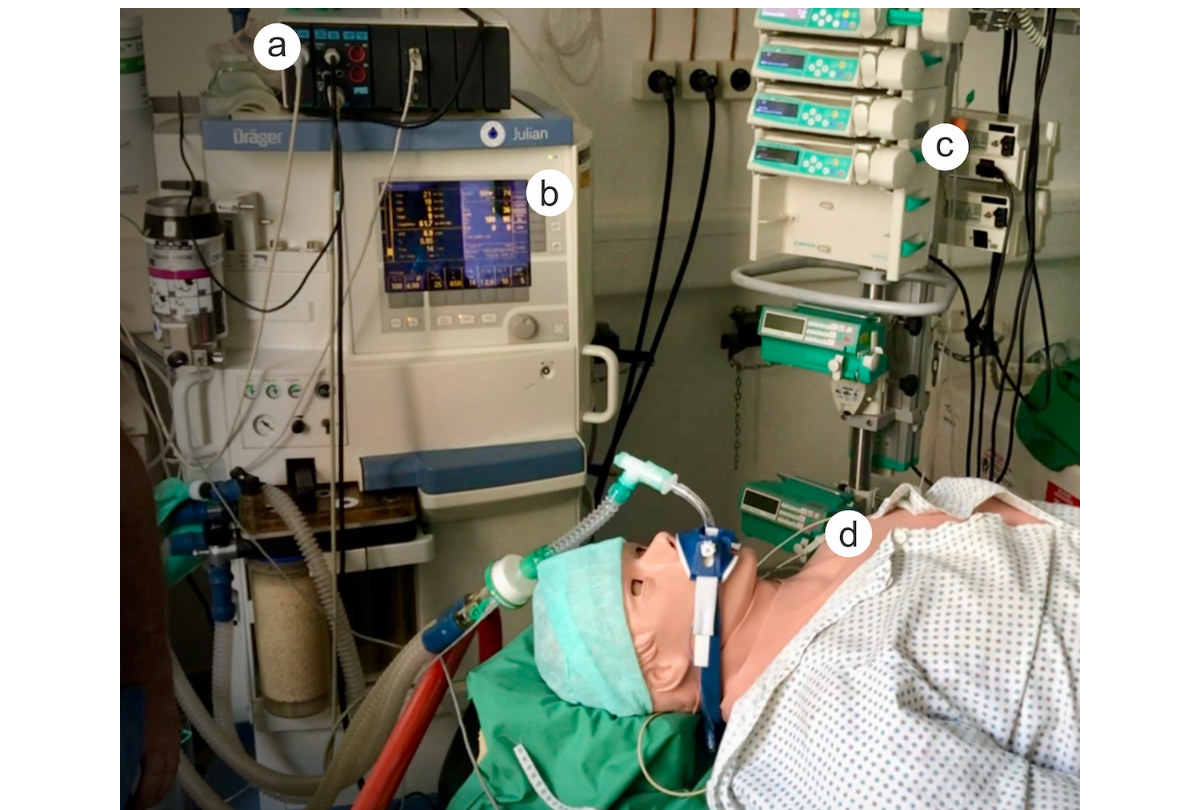
Illustration of the study setup: (a) patient monitor, (b) anesthesia machine, (c) syringe pump, and (d) patient simulator.

In this evaluation, participants used a Julian anesthesia machine (Draeger Medical), a Perfusor Space syringe pump (B Braun), a Datex-Ohmeda AS patient monitor (General Electric), and a filing folder including the printed SSCs for the three operation phases: (1) *sign in*: before induction of anesthesia; (2) *time out*: before incision of the skin; and (3) *sign out*: before the patient leaves the operating room. The SSCs contained the same items as those implemented in the ANWS workflow management. A high-end simulator room at the University Hospital Aachen (AIXTRA) was used to measure the time spans for the same participants carrying out the tasks using conventional machines [23]. For comparison purposes, the response times were measured. Figure 3 illustrates the study setup.

### Study Participants

All 5 subjects—aged 30-42 years; 3 females (60%) and 2 males (40%)—participated in this study voluntarily. The group was composed of anesthesiologists—2 assistant physicians (40%) and 3 consultant anesthesiologists (60%)—from the Department of Anesthesiology of the University Hospital Aachen. The group was heterogeneous regarding practical experience: 3 physicians (60%) had more than 5 years of experience; 1 (20%) had 3-5 years of experience; and 1 (20%) had 1-2 years of experience. None of them had any knowledge of the study scenario or tasks. Only 1 (20%) of the participants had seen the GUI design of the ANWS before, without any connection to the study scenario or tasks. According to the guidelines of the ethics committee, no formal approval was necessary to conduct the study.

### Data Analysis

The primary outcome parameters in this study were the response times and the capability of the subjects in successfully solving each task. As previously discussed, eye tracking was used in this study. The first aim was to identify elements that distracted attention from more important information, known as “vampire effect.” The second aim was to find elements with a hidden affordance. Lastly, eye-tracking information was essential to find areas of interest as well as hitherto unknown impacts or suggestions for improvements.

Using the think-aloud method, it was possible to achieve a better understanding of the participant’s line of thought, reasons for the occurrence of problems or misunderstandings, if applicable, and to identify potential further improvements to the system.

The questionnaire, in turn, was used to track background knowledge and motivation. Information on technical know-how and work experience was requested in order to determine the actual impact of each participant on the study, including a possible halo effect (ie, form of cognitive bias arising from an overgeneralization, such as a limited amount of evidence, the influence of preconceived beliefs, or a priori hypotheses; it is particularly prone to occur among participants who are enthusiastic about technology) [24]. With support from the questionnaire, SUS scores were calculated for the tested features: decision-support system, workflow management, SSC, customizable GUI, and cross-device interaction.

### Statistical Analysis

Statistical analysis was performed using SPSS Statistics for Windows, version 22.0 (IBM Corp). Normal distribution was analyzed with Kolmogorov-Smirnov and Shapiro-Wilk tests. When detecting significance in the comparison of means (ie, between the conventional and OR.NET settings), the Mann-Whitney *U* test and the Wilcoxon signed-rank test were used. A *P* value <.05 was considered statistically significant.

## Results

### Questionnaire: Part A

Table 3 presents the results for the first part of the questionnaire. In general, all 5 participants were enthusiastic about new technologies and used them in their daily life. They classified their technical knowledge as normal or proficient. The great majority (4/5, 80%) agreed that they quickly become used to new technologies and that these make their lives easier. The subjects reported a daily use of mobile phones. The great majority used a tablet (3/5, 60%) and a PC or notebook (4/5, 80%) daily or at least several times a week. Interestingly, some users had already had some private and professional experience with other smart devices, such as smart watches and smart glasses.

**Table 3:** First part of the questionnaire.

Questions and responses		Score^a^, median (min, max)
**Technological use**		
	I often use technical innovation in everyday life.	4 (4, 4)
	I’m skeptical of new technologies.	1 (1, 2)
	I often use technical innovations to make my life easier.	4 (3, 4)
	I quickly get used to using new technologies.	4 (3, 4)
	I can easily use new technologies.	4 (3, 4)
	I don’t like surgical robots in medicine.	1 (1, 2)
	In general, more technology should be used.	4 (2, 4)
	New technologies endanger society.	1 (1, 2)
**I use...**		
	A smartphone (private)	4 (4, 4)
	A smartphone (work)	4 (4, 4)
	A tablet (private)	3 (2, 4)
	A tablet (work)	3 (2, 4)
	A notebook or PC (private)	4 (1, 4)
	A notebook or PC (work)	4 (1, 4)
	Another device; private (eg, smart glasses or smart watch)	1 (1, 4)
	Another device; work (eg, smart glasses or smart watch)	2 (1, 3)
**Study participation**		
	Scientific studies do not support health care delivery and outcomes.	1 (1, 1)
	I expect a compensation or reimbursement for expenses.	1 (1, 3)
	Personally, I do not find questionnaires useful for gathering information on individual or collective perspectives.	1 (1, 3)
	I participate in studies to learn.	3 (2, 4)
**Test conditions**		
	I felt great pressure to perform.	1 (1, 3)
	I felt excessively challenged.	1 (1, 1)
	I was stressed.	2 (1, 2)
	My motivation was high.	4 (3, 4)
	I was focused.	4 (3, 4)

^a^Scores were as follows: 0 (*no statement*), 1 (*disagree*), 2 (*rather disagree*), 3 (*rather agree*), and 4 (*agree*).

All participants (5/5, 100%) agreed that such studies might improve health care delivery and outcomes. In fact, most of the volunteers wanted to participate in order to learn more (4/5, 80%) and did not expect any compensation or reimbursement for expenses (4/5, 80%). In addition, they considered the questionnaires to be helpful for gathering information on individual and collective perspectives.

Regarding the study itself, the participants did not feel pressured, stressed, or challenged. Instead, they were highly motivated and focused in trying to solve the tasks. In fact, 2 physicians (40%) had gained experience in telemedicine through the emergency medical service in Aachen, Germany [25,26]. In addition, 1 (20%) anesthesiologist was involved in configuring and implementing a decision-support system in an intensive care unit.

### Response Time

One of the first tasks in this study consisted of finding the correct SOP. In the ANWS setting, the SOP was automatically generated by the decision-support engine with no delay and was embedded in the workflow management. However, in the conventional setting, the volunteers required 26.8 seconds to accomplish the task.

**Figure 4:**
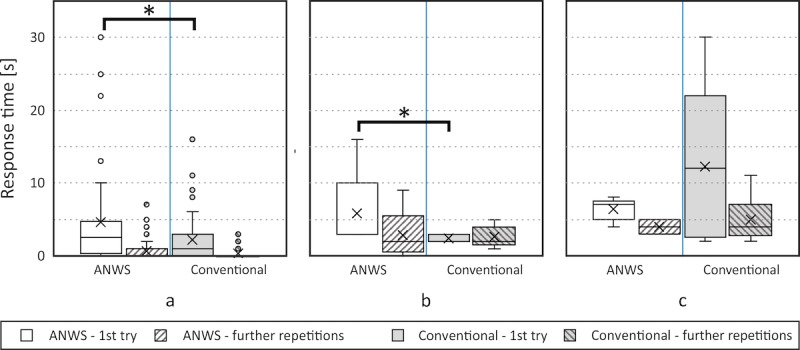
Tukey’s box plots comparing the response times of both groups: anesthesia workstation (ANWS; left) versus conventional setting (right). The “o” represents for outliers, “x” marks the arithmetic mean, and “*” indicates statistical significance (*P*<.05) between the ANWS and conventional groups. To analyze the learning curve, response times for the first try and further repetitions were compared. (a) Time required to read out medical parameters. (b) Time required to set the positive end-expiratory pressure. (c) Time required to set the flow rate of the syringe pump.

As described in Table 1, the subjects were asked in some cases to repeat the same task (ie, nonconsecutive repetitions). In the first round of questions, the time required to read out medical parameters was significantly lower when using the conventional setting (see Figure 4a). The response time was 1 second (interquartile range [IQR] 0-3) for the conventional setting and 2.5 seconds (IQR 0.75-4.25) for the ANWS setting (*P*=.04). A significant decrease in response time was observed directly at the first repetition as depicted in the box plot of Figure 4a. In both phases, it was smaller than 1 second. Interestingly, no difference between groups (ie, phases) was found.

When the subjects were advised to set the PEEP in the anesthesia machine and in the workstation for the conventional and ANWS setting, respectively, the same phenomenon was observed (ie, they required less time when using the conventional approach: conventional, 2 seconds [IQR 2-3]; ANWS, 3 seconds [IQR 3-4]; *P*=.03). The box plot of Figure 4b compares both methods. In the first repetition, the response time for the conventional system did not change: 2 seconds (IQR 2-3). In turn, a decrease in response time for the ANWS setting was observed: 2 seconds (IQR 1-2). Here, the *P* value was .52. In this case, a learning effect with decreasing response time was observed for the ANWS: 3 seconds (IQR 3-4) versus 2 seconds (IQR 1-2; *P*=.35). This was not observed for the conventional setting: 2 seconds (IQR 2-3) versus 2 seconds (IQR 2-3; *P*=.66).

As represented in Figure 4c, the same trend was not visible when setting the flow rate of the syringe pump. In the first try, the subjects accomplished the task within 7 seconds (IQR 6-7) and 12 seconds (IQR 3-14; *P*=.60) using the ANWS and the conventional system, respectively. As expected, the response time decreased significantly in further repetitions, especially for the conventional setting; the median was 4 seconds (IQR 3-6.75) for the conventional setting and 4 seconds (IQR 3-4.75) for the ANWS setting (*P*=.72).

During the implantation of prosthetic sockets in total hip arthroplasties and in surgeries in general, several complications may occur. Within the surgery simulation, a pulmonary embolism was mimicked (see Table 1). The intelligent alarm, integrated in the ANWS, notified the anesthesiologist immediately about the potential occurrence of this particular complication due to changes in vital signs. During the conventional trial, 6.8 seconds (IQR 2-10) elapsed before participants detected the complication.

### Eye Tracking and the Think-Aloud Method

All areas of interest were identified by each participant. However, the usability study demonstrated that improvements in some elements of the GUI were still necessary. When starting the surgery workflow, the button used to select the patient and the corresponding operation, in this case a total hip arthroplasty, showed a clear case of hidden affordance. Furthermore, it was not intuitively obvious which button needed to be clicked to update the workflow process. Although eye movements indicated searching and the participants often looked directly at the correct button, they had difficulties in understanding its purpose. Another significant case of hidden affordance was recognized when dragging and dropping the device panels from the device selector (see Figure 1b) to the central region of the GUI (see Figure 1d). Otherwise, no “vampire effects” were detected.

### Questionnaire: Part B

**Figure 5:**
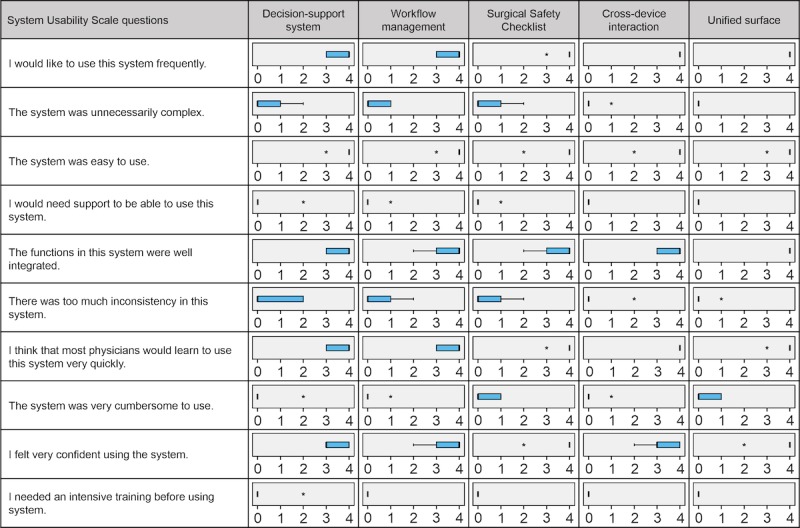
The second part of the questionnaire: the System Usability Scale results presented by Tukey’s box plots. Each feature was rated from 0 (*strongly disagree*) to 4 (*strongly agree*). Asterisk indicates an outlier.

Figure 5 shows the evaluation of a segment of the questionnaire. In general, a good acceptance of the ANWS functional model was observed. In fact, in the SUS, all features were graded as at least *excellent* as follows: decision-support system, 95 points (IQR 87.5-97.5); workflow management, 97.5 points (IQR 82.5-97.5); SSC, 100 points (IQR 87.5-100); cross-device interaction, 97.5 points (IQR 92.5-100); and unified surface, 100 points (IQR 92.5-100).

**Figure 6:**
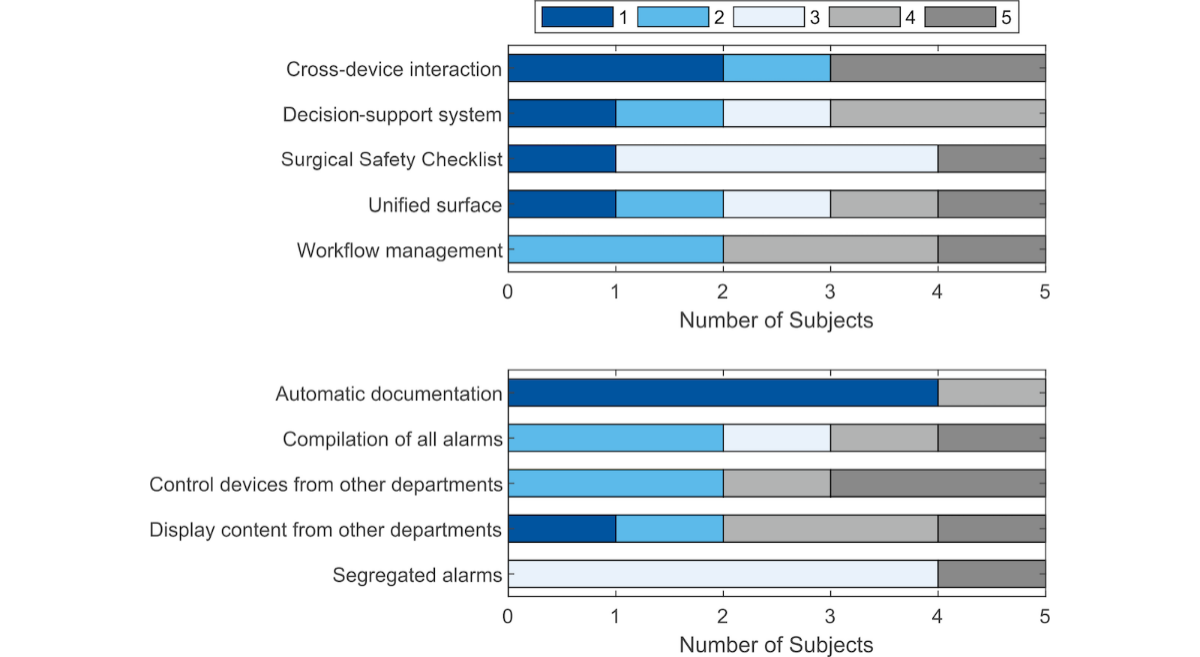
Tested (top) and untested (bottom) features were rated by the subjects from 1 (*favorite,* dark blue) to 5 (*least favorite,* dark gray).

In the questionnaire, the volunteers were also asked to sort ANWS features—tested and untested—according to their preference, from 1 (*favorite*) to 5 (*least favorite*). Figure 6 (top and bottom) shows the results for tested and untested features, respectively. Regarding the tested features, no unanimity was observed (ie, the preference varied between the subjects). Although the subjects did not test the feature *automatic documentation*, the great majority found it meaningful (see Figure 6). *Compilation of all alarms* and *control devices from other departments* were the second-favorite features of the anesthesiologists.

**Figure 7:**
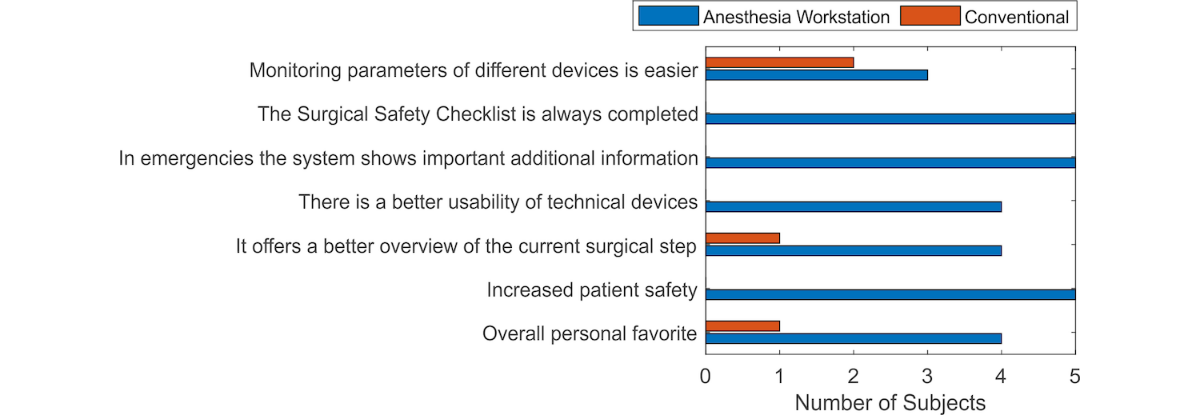
Graphical representation of the subjects’ favorite system, anesthesia workstation or conventional systems, according to seven criteria.

At the end of the questionnaire, the subjects were asked to choose, for each criterion, their favorite system: ANWS or conventional. The results, represented in Figure 7, demonstrated that they favored the ANWS functional model. In their opinion, it would permit them to do the following:

1. Better monitor the parameters of different devices.
2. Always complete the SSC.
3. Get important additional information.
4. Improve the overall usability of the connected devices.
5. Get a better overview of the current surgical step.
6. Increase patient safety.

In general, the subjects demonstrated clear opinions with the exception of subject 2; he had a neutral opinion with regard to item 4. Although the majority of the subjects considered the ANWS functional model as their personal favorite system, 2 out of 5 (40%) considered the use of the ANWS to be more complicated when rating the *monitoring of parameters from different devices is easier* criterion. Furthermore, during an emergency surgery, 4 out of 5 doctors (80%) would prefer to use the conventional system.

## Discussion

### Principal Findings

In this study, 5 anesthesiologists were requested to test and evaluate a newly self-developed ANWS functional model. To test its usability, the subjects were requested to perform several tasks using the ANWS and the conventional system. The ANWS was deemed noninferior compared to the well-known system that was used daily.

All subjects considered themselves *enthusiastic about new technologies* and their technical knowledge ranged between *normal* and *proficient*. This was confirmed during the study. In general, they did not demonstrate having difficulties while solving the tasks with the ANWS functional model and accomplished them successfully.

To analyze the learning curve, some tasks were repeated. As expected, during the first try, the subjects requested more time (2.5 seconds [IQR 0.75-4.25] vs 1 second [IQR 0-3]) when using the ANWS. During further repetitions, the response time decreased significantly, resulting in a time span of less than 1 second in each setting. As demonstrated in Figure 4, the response times during further repetitions were very similar for the conventional and ANWS settings. These results suggest that, before using the new functional model, initial training is necessary. However, they also show a steep learning curve for physicians with normal-to-proficient technical knowledge. In this study, we also observed that this learning curve is similar for other medical devices. One of the tasks consisted of manually setting the flow rate of a syringe pump. Since some of the participants were not familiar with operating this specific device, greater response times were observed in the first try. Once more, this emphasizes that for any medical device, initial training should be mandatory.

Unfortunately, hospitals frequently utilize medical devices from various manufacturers, even for the same appliance classes, such as patient monitoring, ventilators, and syringe pumps, which means different modus operandi. Consequently, the physician must be capable of working with all of them and of changing over without any time delay. As a result, *information overload* commonly leads to user errors; this can be ameliorated by medical device interoperability. Toward this end, a unified GUI would be especially beneficial in order to reduce (1) the number of training sessions, (2) response times, (3) potential use errors, and (4) medical costs.

Figure 4a contains a few outliers for the ANWS and the conventional system. A data point is considered an outlier if it exceeds or is 1.5 times the IQR above the 3rd quartile or 1.5 times the IQR below the 1st quartile. Due to the relatively low number of participants, outliers are expected; even so, no significant difference between the conventional and ANWS settings occurred. In each system, 40 data points for the first try and 70 data points for further repetitions were collected.

Another important advantage of the ANWS is the fact that the World Health Organization SSC is integrated into the surgical workflow panel so that it must be filled in. Therefore, this feature contributes to improved patient safety and quality of care since SSCs are often neglected in clinical practice. In addition, the functional model also includes a decision-support system aimed at assisting the medical team in the decision-making process. It uses patient data (eg, vital parameters) as input measures and combines them with mathematical models and algorithms. When potential abnormal changes are detected, the physicians are informed (eg, by an alarm). As demonstrated in the Response Time section of the Results, the anesthesiologists required more time to detect the pulmonary embolism when using the conventional system. In contrast, when using the ANWS, the subjects were promptly informed about this complication and could start with the treatment directly. Of course, the decision-support system does not replace the physician, but is able to support him or her and, therefore, improve the quality of care.

Regarding the questionnaire, the subjects considered automatic documentation a very important feature since it contributes to a reduction in the workload. Concerning the remaining features, no consensus was reached because preferences are usually subjective and vary from individual to individual. However, in general, a great acceptance of the ANWS functional model was observed. Indeed, all tested features were graded as at least *excellent*. The majority of subjects agreed that the ANWS might permit the monitoring of parameters of different devices in a more effective and efficient way and allow physicians to do the following: always complete the SSC, get important additional information, improve the overall usability and safety within the usage of the connected devices, get a better overview of the current surgical step, and increase patient safety. Based on these reasons, they chose the functional model as their personal favorite. Despite that, 4 out of 5 subjects (80%) would still prefer to use the conventional system. This is reasonable, since the ANWS is neither ready for market nor clinically approved. In addition, more meticulous training would be necessary. Conversely, 1 subject (20%) stated they would use the ANWS straight away. This unexpected answer probably indicates a halo effect.

Despite the positive feedback, there is still room for improvement. Taking the eye-tracking and think-aloud method analyses into account, two controls indicated hidden affordance: the workflow management controls and the drag-and-drop mechanism of the device panels need to be adapted to improve usability. Fortunately, no “vampire effects” were detected and all areas of interest were identified clearly.

### Limitations

This study has certain limitations, which need to be addressed. First, due to the early stage of development of the ANWS, this pilot study was carried out with only 5 participants, which is the minimum requirement for formative usability tests within the usability engineering process for medical devices, according to the International Electrotechnical Commission (IEC) 62366 standard. Therefore, the statistical capacity is still limited for this pilot study.

Second, not all features of the functional model were investigated (eg, automatic documentation). Third, the experimental settings were not very close to reality, especially when considering the ANWS testing. Due to the lack of SDC-compatible devices, all measures were simulated digitally but were not derived from a patient or a patient simulator. Finally, the functional model is not certified as medical device, hence, a simulator instead of *real human subjects* was used to provide the data for the ANWS.

Despite these drawbacks, this study is, to the best of our knowledge, the first analysis regarding the acceptance of an innovative and pioneering ANWS, based on a manufacturer-independent communication protocol, namely SDC.

Although the ANWS only turned out to be noninferior compared to the conventional setting, this is already promising. We believe that the ANWS would have been superior if the following had been provided:

1. Adequate training of the professional users, analogous to the conventional system.
2. Optimization of the GUI in terms of usability aspects and design.
3. Integration into a clinical environment including *real* patient-derived measures.
4. Actual tasks during real anesthesiologic workflows, such as anesthesia induction, maintenance, and complications.
5. Complex scenarios that benefit from *smart alarms* and decision-support engines.

### Conclusions

Although the technologizing of hospitals, especially in operation theaters, is increasing, medical devices and IT systems mainly work as stand-alone versus networked systems. Consequently, progress in health care digitization is very slow. In this project, we developed an anesthesia dashboard, enabling various sophisticated features based on open device interconnection. In the meantime, our project team contributed to the development and approval of SDC, a manufacturer-independent IEEE 11073 standard for medical device networking, which was approved as a worldwide accepted standard in January 2019.

The way is now paved for manufacturers to equip their medical devices with SDC-compliant interfaces, enabling interconnectivity in the operation theater and elsewhere in health care.

## Abbreviations

ANWS: anesthesia workstation
GUI: graphical user interface
HCI: human-computer interaction
IEC: International Electrotechnical Commission
IEEE: Institute of Electrical and Electronics Engineers
IQR: interquartile range
OR.NET: Secure Dynamic Networking in the Operating Room and Clinic
OSCLib C#: OpenStackClient C# library
PEEP: positive end-expiratory pressure
SDC: service-oriented device connectivity
SOP: standard operating procedure
SSC: Surgical Safety Checklist
SUS: System Usability Scale

## References

[1] Checketts MR, Alladi R, Ferguson K, Gemmell L, Handy JM, Klein AA, Love NJ, Misra U, Morris C, Nathanson MH, Rodney GE, Verma R, Pandit JJ, Association of Anaesthetists of Great Britain and Ireland (2016). Recommendations for standards of monitoring during anaesthesia and recovery 2015: Association of Anaesthetists of Great Britain and Ireland. Anaesthesia.

[2] Imhoff M, Fried R (2009). The crying wolf: Still crying?. Anesth Analg.

[3] Kasparick M, Golatowski F, Timmermann D (2017). A safe and interoperable distributed alarm notification system for PoC medical devices using IEEE 11073 SDC. Proceedings of the 2017 IEEE Healthcare Innovations and Point of Care Technologies (HI-POCT).

[4] Faiola A, Srinivas P, Duke J (2015). Supporting clinical cognition: A human-centered approach to a novel ICU information visualization dashboard. AMIA Annu Symp Proc.

[5] Koeny M, Benzko J, Czaplik M, Walter M, Radermacher K, Rossaint R, Leonhardt S (2012). Getting anesthesia online: The smartOR network. Int J Adv Internet Technol.

[6] Koeny M, Leonhardt S, Czaplik M, Rossaint R (2012). On the road to predictive smart alarms based on a networked operating room. Proceedings of the 25th IEEE International Symposium on Computer-Based Medical Systems (CBMS).

[7] Koeny M, Czaplik M, Walter M, Rossaint R, Leonhardt S (2011). A new telesupervision system integrated in an intelligent networked operating room. Proceedings of EMERGING 2011, The Third International Conference on Emerging Network Intelligence.

[8] Balust J, Macario A (2009). Can anesthesia information management systems improve quality in the surgical suite?. Curr Opin Anaesthesiol.

[9] Shieh JS, Linkens DA, Peacock JE (1999). Hierarchical rule-based and self-organizing fuzzy logic control for depth of anaesthesia. IEEE Trans Syst Man Cybern C Appl Rev.

[10] Fan SZ, Yeh JR, Chen BC, Shieh JS (2011). Comparison of EEG approximate entropy and complexity measures of depth of anaesthesia during inhalational general anaesthesia. J Med Biol Eng.

[11] Birkle M, Benzko J, Shevchenko N (2012). Das Projekt OR.NET: Sichere dynamische vernetzung in OP und klinik [The OR.NET Project: Secure dynamic networking in the OR and clinic]. Dtsch Z Klin Forsch, Innov Praxis.

[12] Kasparick M, Schlichting S, Golatowski F, Timmermann D (2015). Medical DPWS: New IEEE 11073 standard for safe and interoperable medical device communication. Proceedings of the 2015 IEEE Conference on Standards for Communications and Networking (CSCN).

[13] Mildner A, Janß A, Dell'Anna-Pudlik J, Merz P, Leucker M, Radermacher K (2015). Device- and service profiles for integrated or systems based on open standards. Curr Dir Biomed Eng.

[14] Kasparick M, Schmitz M, Andersen B, Rockstroh M, Franke S, Schlichting S, Golatowski F, Timmermann D (2018). OR.NET: A service-oriented architecture for safe and dynamic medical device interoperability. Biomed Tech (Berl).

[15] Kasparick M, Schmitz M, Golatowski F, Timmermann D (2016). Dynamic remote control through service orchestration of point-of-care and surgical devices based on IEEE 11073 SDC. Proceedings of the IEEE Healthcare Innovation Point-Of-Care Technologies Conference (HI-POCT).

[16] Andersen B, Kasparick M, Ulrich H, Franke S, Schlamelcher J, Rockstroh M, Ingenerf J (2018). Connecting the clinical IT infrastructure to a service-oriented architecture of medical devices. Biomed Tech (Berl).

[17] Czaplik M, Voigt V, Kenngott H, Clusmann H, Hoffmann R, Will A, Further members of the Medical Board‚ BMBF Research Project “OR.NET – Secure Dynamic Networking in the Operating Room and Clinic” (2018). Why OR.NET? Requirements and perspectives from a medical user's, clinical operator's and device manufacturer's points of view. Biomed Tech (Berl).

[18] Duchowski AT (2007). Eye Tracking Methodology: Theory and Practice. 2nd edition.

[19] Holzinger A (2005). Usability engineering methods for software developers. Commun ACM.

[20] Kuvita T, Karlíček M (2014). The risk of vampire effect in advertisements using celebrity endorsement. Cent Eur Bus Rev.

[21] Jordan PW, Thomas B, Weerdmeester BA, Clelland IL (1996). Usability Evaluation in Industry.

[22] Bangor A, Kortum P, Miller J (2009). Determining what individual SUS scores mean: Adding an adjective rating scale. J Usability Stud.

[23] Krüger A, Gillmann B, Hardt C, Döring R, Beckers SK, Rossaint R (2009). Teaching non-technical skills for critical incidents: Crisis resource management training for medical students [Article in German]. Anaesthesist.

[24] Nisbett RE, Wilson TD (1977). The halo effect: Evidence for unconscious alteration of judgments. J Pers Soc Psychol.

[25] Skorning M, Bergrath S, Rörtgen D, Brokmann JC, Beckers SK, Protogerakis M, Brodziak T, Rossaint R (2009). E-health in emergency medicine: The research project Med-on-@ix [Article in German]. Anaesthesist.

[26] Felzen M, Brokmann JC, Beckers SK, Czaplik M, Hirsch F, Tamm M, Rossaint R, Bergrath S (2017). Improved technical performance of a multifunctional prehospital telemedicine system between the research phase and the routine use phase: An observational study. J Telemed Telecare.

